# Novel Strategies for Upstream and Downstream Processing of Tannin Acyl Hydrolase

**DOI:** 10.4061/2011/823619

**Published:** 2011-09-19

**Authors:** Luis V. Rodríguez-Durán, Blanca Valdivia-Urdiales, Juan C. Contreras-Esquivel, Raúl Rodríguez-Herrera, Cristóbal N. Aguilar

**Affiliations:** ^1^Food Research Department, School of Chemistry, Autonomous University of Coahuila, Boulevard V. Carranza and González Lobo s/n, 25280 Saltillo, Coahuila, Mexico; ^2^Research and Development Center, Coyotefoods Biopolymer and Biotechnology Co., Simón Bolívar 851-A, 25280 Saltillo, Coahuila, Mexico

## Abstract

Tannin acyl hydrolase also referred as tannase is an enzyme with important applications in several science and technology fields. Due to its hydrolytic and synthetic properties, tannase could be used to reduce the negative effects of tannins in beverages, food, feed, and tannery effluents, for the production of gallic acid from tannin-rich materials, the elucidation of tannin structure, and the synthesis of gallic acid esters in nonaqueous media. However, industrial applications of tannase are still very limited due to its high production cost. Thus, there is a growing interest in the production, recovery, and purification of this enzyme. Recently, there have been published a number of papers on the improvement of upstream and downstream processing of the enzyme. These papers dealt with the search for new tannase producing microorganisms, the application of novel fermentation systems, optimization of culture conditions, the production of the enzyme by recombinant microorganism, and the design of efficient protocols for tannase recovery and purification. The present work reviews the state of the art of basic and biotechnological aspects of tannin acyl hydrolase, focusing on the recent advances in the upstream and downstream processing of the enzyme.

## 1. Introduction

Tannin acyl hydrolase also known as tannase is an enzyme (EC 3.1.1.20) that catalyzes the hydrolysis of ester bonds present in gallotannins, complex tannins, and gallic acid esters [1, 2]. Tannase has several important applications in food, feed, chemical and pharmaceutical industries, but high scale use of this enzyme is severally restricted due to high production costs [3, 4].

Thus, there is a growing interest on basic and applied aspects of tannase. In the last decade, there have been a number of efforts to improve the production, recovery, and purification processes of the enzyme. These efforts include the looking for new tannase sources [5–12], the development of novel fermentation systems [13–15], the optimization of culture conditions [16–19], the production of the enzyme by recombinant microorganism [20–24], and the design of efficient protocols for tannase recovery and purification [25–27].

Technological advances on tannase processing must be supported by basic investigation. The induction and repression systems have been studied in submerged- (SmF) and solid-state fermentation systems (SSF) [28, 29]; the physicochemical properties of several tannases have been characterized [7, 12, 20, 30–35], and there have been a special interest in the description of tannase and tannase gene structure [21, 36–44]. 

The present work reviews the state of the art of basic and biotechnological aspects of tannin acyl hydrolase, focusing on the recent advances in the upstream and downstream processing of the enzyme.

## 2. Tannase Substrate: Tannins

Tannins are natural polyphenolic compounds that are widely distributed in several parts of vascular plants. They are the second most abundant group of phenolics in nature and are considered as secondary metabolic compounds of plants because they play no direct role in plant metabolisms [9]. Tannins are characterized by their ability to form strong complexes with different minerals and macromolecules, such as proteins, cellulose, and starch among others [45]. Due to their strong ability to bind with proteins, they have been used for tanning for thousands of years [46]. 

Tannins have several interesting biological activities. Active principles of medicinal plants are often polyphenolic compounds, and, in recent years, there has been a great scientific interest for this group of compounds due to their antioxidant, antiviral, and anticancer properties [47, 48]. In traditional medicine of China and Japan, the plant extracts rich in tannins have been used as astringent, anti-diarrheal, diuretic, anti-inflammatory, antiseptic, and anti-hemorrhagic agents. Similarly for their ability to precipitate heavy metals and some alkaloids, tannins can be used in the treatment of poisoning caused by these substances [49].

In the other hand, presence of tannins in the diet of ruminants affects their growth and milk production in different ways. Interaction of tannins with macromolecules form complexes that interfere with digestion of certain nutrients, digestive enzymes in saliva and rumen, and thereby reduce the degradation of nutrients. In addition, tannins impart a bitter taste, and this could significantly reduce the feed intake by livestock [50, 51]. 

High concentrations of tannins in beverage such as iced tea, beer, wine, fruit juices, and coffee-flavored beverages can result in the formation of precipitates due to their interaction with other molecules present in these beverages. These undesirable effects of tannins can be reduced or eliminated by a chemical or enzymatic treatment [50, 52]. 

Tannins are resistant to biodegradation, and the accumulation due to discharge of tanneries and coffee-processing industries effluents can result in environmental pollution [53, 54]. Tannins are toxic to fungi, bacteria, and viruses. However, many microorganisms have developed the mechanisms to overcome the effects of tannins. These mechanisms include tannin modification, degradation, dissociation of tannin-substrate complexes, tannin inactivation by high-affinity binders, membrane modification, and metal ion sequestration [8]. Tannase is involved in the biodegradation of tannins and is an ecologically important biocatalyst.

## 3. Basic Aspects of Tannin Acyl Hydrolase

As previously mentioned, tannase catalyzes the hydrolysis of ester and depside bonds present in gallotannins, complex tannins, and gallic acid esters [1, 2] but do not affect the carbon-carbon bonds, thus tannase is unable to hydrolyze condensed tannins [55].

It is well known that tannase catalyzes the hydrolysis of tannic acid (nonagalloyl glucose) to nine molecules of gallic acid and one molecule of glucose, but the mechanism and the intermediary compounds are not clearly understood. 

### 3.1. Substrate Specificity

It has been a matter of dispute whether the esterase and depsidase activities of microbial tannases were due to two separate enzymes or to only one enzyme catalyzing both reactions [56]. Toth and Barsony [57] proposed that tannase activity is composed by two separated enzymes: a “depsidase” that hydrolyzes the depside bonds (galloyl ester of gallic acid) and an “esterase” that catalyzes the cleavage of ester (galloyl ester of an alcohol moiety). Later, Haslam and Stangroom reported that the esterase/depsidase ratio of *Aspergillus niger *tannase may be modified by cultural methods and isolation procedures, suggesting the presence of two different enzymes. But further analysis indicated that esterase and depsidase are isoenzymes with low specificities capable of hydrolyzing both esters and depsides of gallic acid but with different relative specificity for each substrate [55]. Beverini and Metche [58] isolated two separate isoenzymes, tannase I and tannase II from *A. oryzae*, with esterase and depsidase activity, respectively. But several other authors have purified tannases with both esterase and depsidase activity [20, 21, 59].

Studies on the regulation of tannase indicated that the enzyme would react with any phenolic hydroxyl group [60]. But, for the formation of a true enzyme-substrate complex, the substrate has to be an ester compound of gallic acid, although there is no restriction on the structure of an alcohol composing a substrate ester. These studies also indicated that the ester or carboxyl does not link to the enzyme by itself, since an ester or carboxylic compound is not hydrolyzed by or inhibits the enzyme unless it has phenolic hydroxyls [24]. Further studies revealed that esters of protocatechuic acid (3,4-dihydroxybenzoic acid) can also be hydrolyzed by tannase [20, 61]. An exceptional case is a tannase analogue extracted from pedunculate oak (*Quercus robur*) leaves. This plant tannase is an unspecific esterase, capable of hydrolyzing simple galloyl esters (methyl, ethyl, and propyl gallate). naphthyl acetate, mono- to hexa-substituted galloyl-*β*-D-glucoses, variously ring-substituted 1-*O*-benzoyl-*β*-D-glucoses and with depsides like *meta*-digallic acid or chlorogenic acid [56]. 

On the other hand, a few bifunctional tannases have been reported. Ramírez-Coronel and coworkers found an *Aspergillus niger *tannase with sequence similarity to an *A. kawachi *β**-glucosidase. Purified tannase was capable to hydrolyze cellobiose efficiently. However, no *β*-glucosidase activity was detected when the enzyme was assayed in the presence of tannic acid [42]. García-Conesa et al. [62] found an *A. oryzae *capable to hydrolyze several synthetic diethyl diferulates. The efficiency of this esterase activity on most diferulates is low compared to that of a cinnamoyl esterase, FAEA, from *A. niger*.

Summarizing, tannases are a group of esterases with more or less specificity for a substrate. This specificity depends on the source and the methods utilized for its production and isolation. Additionally, it is evident that, from a certain organism, several tannase isoenzymes with different substrate affinity could be isolated.

### 3.2. Mechanism of Action

**Figure 1:**
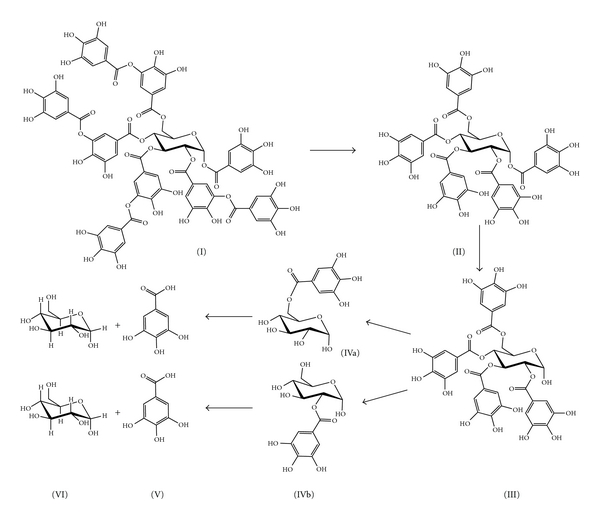
Tannic acid hydrolysis pathway as proposed by Iibuchi et al. [60].

Tannase completely hydrolyzes tannic acid to gallic acid and glucose. Iibuchi et al. [60] studied the intermediary compounds formed during this hydrolysis by thin layer chromatography. They found the formation of 2,3,4,6-tetragalloylglucose, two kinds of monogalloylglucose and free gallic acid. They detected the same products in the hydrolysate of 1,2,3,4,6,-pentagalloylglucose. With this information they proposed a degradation pathway (Figure 1).

**Figure 2:**
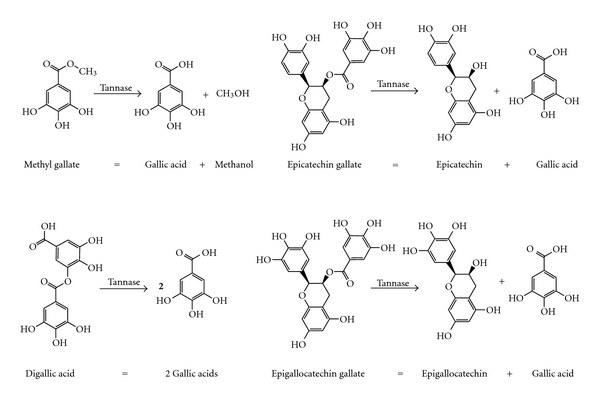
Several hydrolytic reactions catalyzed by tannase.

**Figure 3:**
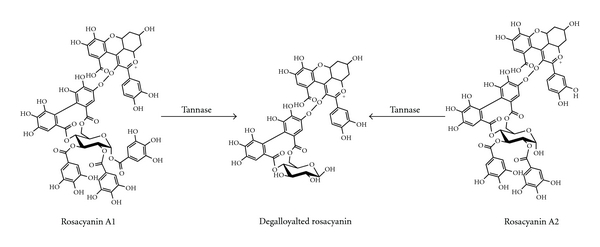
Selective hydrolysis of galloyl moieties of ellagitannins catalyzed by tannase.

Tannase hydrolyzes other substrates such as methyl gallate, propyl gallate, digallic acid, epicatechin gallate, and epigallocatechin gallate-releasing gallic acid [20, 63]. Tannase also acts on ellagitannins such as rosacyanin or phyllanemblinin. In those cases, tannase selectively hydrolyses the galloyl moieties, yielding gallic acid and degalloylated ellagitannins [64, 65]. These reactions are illustrated in Figures 2 and 3.

### 3.3. Physicochemical Properties

One of the most studied topics on tannase is that related to its physicochemical properties. Several fungal, bacterial, and plant tannases have been purified and characterized. Important differences have been found between characterized tannases. These differences are related to the organism that produced the enzyme, the source of the organism, and the production conditions [4]. 

All known tannases are serine esterases, as inferred from inhibition studies with phenyl-methyl-sulfonyl fluoride (PMSF) and diisopropyl fluorophosphate (DFP) [31, 32, 37, 59] and the presence of the pentapeptide motif (-Gly-X-Ser-X-Gly-) in the sequence of tannase gene [21, 66]. However, literature reveals that the protein tannase is very diverse in its structural properties (Table 1).

 The molecular weight of characterized tannases was found to be in the range of 50–320 kDa [21, 32, 40, 43, 69] depending on the source. Most of fungal tannases have been reported to be multimeric proteins formed by 2 to 8 subunits. For example, Ramírez-Coronel et al. [42] purified and characterized an *Aspergillus niger* tannase which is active in monomeric and dimeric isoforms of 90 and 180 kDa, respectively; Böer and coworkers found that tannase from the dimorphic yeast *Arxula adeninivorans *is composed homotetramer with subunits of 80 kDa [21]; Beena et al. reported a tannase of *A. awamori* formed by six identical subunits of 37.8 kDa [37]; otherwise, Hatamoto et al. reported that native tannase of *A. oryzae* consists of four pairs of two types of subunits (30 and 34 kDa, resp.) linked together by disulfide bonds, forming a heterooctamer of 310 kDa [43]. Furthermore, all bacterial tannases characterized are monomeric with a molecular weight ranging from 50 to 90 kDa [39, 40, 68].

All fungal and yeast tannases are glycoprotein with a variable content of carbohydrate ranging from 5.4 to 64% [12, 21, 37, 58, 59, 67, 70, 71]. On the contrary, bacterial tannases seems not to present such posttranslational modifications [40, 68]. Tannase glycosylations consist primarily of neutral sugars like mannose, galactose, and hexosamines [24]. 

The biological function of this high carbohydrate content is unknown but may be related to ability to tolerate the denaturing action of tannin [52]. The carbohydrate coating probably protects the polypeptide backbone, which would then be less accessible to tannin molecules. Consequently, binding of tannin by glycoprotein probably occurs with the carbohydrate rather than with the protein, perhaps creating a weaker and more readily reversible complex. This hypothesis is supported by the observation that tannase and other tannin-resistant proteins are glycoprotein with a high content of carbohydrate [72].

The above variation in the tannase structure can be attributed to the presence of various isoforms of the enzyme, or it may be also due to the presence of signal sequences which are required to transport the enzyme molecule from the cytosolic part of cell to the outside [4, 39].

Despite the structural differences between the tannases known, there are some physicochemical properties that remain more or less similar, as shown in Table 2. Most of tannases have been reported to have optimal temperature of activity between 30 and 40°C [3]. However, there are some information on the characterization of psychrophilic or thermophilic tannases; for example, Ramírez-Coronel et al. [42] and Battestin et al. [35] reported the production of thermo-stable tannases by *Aspergillus niger *and *Paecilomyces variotii *in SSF; these enzymes have their optimal activity at 70°C. On the other hand, Kasieczka-Burnecka et al. [12] purified and characterized two psychrophilic tannases from an Antarctic strain of *Verticillium* sp., with an optimal temperature of 20 and 25°C, respectively.

In the case of optimum pH, most of the studied tannases showed their maximum activity at acid pH values (4.3–6.5), with isoelectric point ranged from 4.3 to 5.1 in most of the cases and are found to be stable in a wide range of pHs (2.0–8.0) [24, 59, 73, 77–79]. Recently, it has been reported that several tannases are highly active in extreme conditions of pH. Beena et al. [37] characterized an acidophilic tannase with optimal activity at pH 2.0; also the enzyme retained around 80% of its maximal activity at pH 1.0. On the other hand, the tannase of *Lactobacillus plantarum* ATCC 14917 has an optimal pH of 8.0 and retains about 88% its maximal activity at pH 9.0 [40]. 

It has been reported that the *K_M_* value of tannase for different fungi with tannic acid was different. The *K_M_* values were 0.28, 0.95, 1.05, 0.048, and 0.00061 for tannase of *A. niger*, *Cryphonectria parasitica*, *Verticillium* sp., *Penicillium chrysogenum*, and *Paecilomyces variotii*, respectively [12, 79–81].

### 3.4. Tannase Gene

Hatamoto et al. [43] first reported the complete sequence of a tannase gene. They found the absence of introns in *Aspergillus oryzae* tannase gene and that it coded for a sequence of 588 aminoacids with a molecular weight of about 64000 kDa. Analysis of native protein indicated that the tannase gene product is translated as a single polypeptide that is cleaved by posttranslational modification into two tannase subunits linked by a disulfide bond. They concluded that mature protein consisted of four pairs of the two subunits, forming a hetero-octamer with a molecular weight of about 300 000. Since then, the tannase gene of a number of organisms has been identified by structural homology, but only a few have been confirmed at protein level. 

Recently, Leόn-Galván and coworkers [38] cloned and sequenced the complete cDNA of a tannase gene from *Aspergillus niger*. The open reading frame (ORF) was found to be of 1833 bp. The 5′ untranslated (UTR) region consisted of 1822 bp and a 3′ UTR of 1015 bp; both regions are substantially larger than the previously reported for the tannase gene of *A. oryzae*. Homology analysis of tannase ORF displayed a 75% identity with *A. oryzae*. Beena et al. [37] isolated and sequenced the tannase gene from a marine *A. awamori *strain and found an ORF of 1,122 bp. Homology studies revealed a higher similarity of the *A. awamori* gene with *A. niger* gene (82% identity) than with the *A. oryzae* gene (77%). Böer et al. [21] identified the tannase-encoding gene from the dimorphic yeast *Arxula adeninivorans. *The gene has an ORF of 1764 bp and encodes a 587-amino acid protein, preceded by an N-terminal secretion sequence comprising 28 residues. The deduced amino acid sequence was similar to those of tannases from *A. oryzae* (50% identity) and *A. niger* (48%).

**Figure 4:**
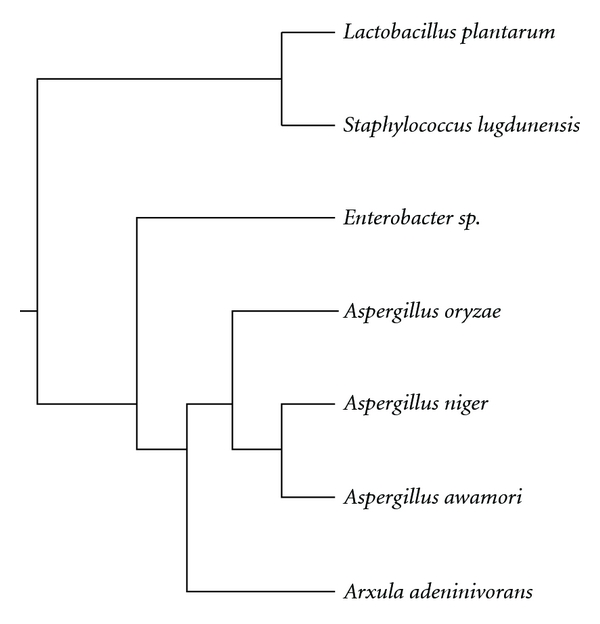
Phylogenetic tree showing the relationship between the reported sequences of the tannase-encoding gene.

On the other hand, Noguchi et al. [36] first reported a tannase gene from bacteria. They cloned and sequenced a novel gene (*tanA*) from *Staphylococcus lugdunensis* that encodes a polypeptide of 613 amino acids with tannase activity. The *tanA* gene was found to be specific for *S. lugdunensis* and has no significant similarity with genes of fungal tannases [82]. Later, Iwamoto and coworkers cloned and sequenced the tannase gene from *Lactobacillus plantarum* (*tanLpl*). The *tanLpl* gene was almost identical to a nucleotide sequence of *L. plantarum* WCFS1 designated as lp2956 (99.6% identity), encoding a hypothetical protein but with a single base substitution at four positions and was similar (46.7%) to *tanA* from *S. lugdunensis *[40]. More recently, Sharma and John [39] reported the characterization of the tannase gene from *Enterobacter* sp. Multiple alignment showed that *Enterobacter* sp. Tannase is not very much similar to tannase of *S. lugdunensis* and *L. plantarum*, since only 10% and 13% amino acid residues of *Enterobacter* sp. tannase are similar to those of *S. lugdunensis* and *L. plantarum* tannases, respectively. Additionally, bacterial tannase genes are not closely related to fungal tannases, as shown in Figure 4.

## 4. Applications of Tannin Acyl Hydrolase

Tannase has several interesting applications in food, feed, chemical, and pharmaceutical industries. At the moment, the principal uses of tannase are in the elaboration of instantaneous tea and the production of gallic acid ester by depolymerization of tannin-rich materials [52]. But, due its hydrolytic and synthetic properties, tannase has several other potential applications. Several patents on these uses of tannases are showed in Table 3.

### 4.1. Instantaneous Tea Elaboration

After water, tea is the second most highly consumed beverage worldwide [83]. It is an infusion obtained from leaves of *Camellia sinensis* and is consumed by two-thirds of the world's population [84]. Tea drinking is associated with the reduction of serum cholesterol, prevention of low-density lipoprotein oxidation, and decreased risk of cardiovascular disease and cancer [85]. 

During the production of tea beverages, hot and clear tea infusions tends to form turbid precipitates after cooling. These precipitates, called tea cream, are formed by a complex mixture of polyphenols. Tea cream formation is a quality problem and may have antinutritional effects [86]. Tannase can hydrolyze the ester bonds of catechins to release free gallic acid and water-soluble compounds with lower molecular weight, reducing turbidity and increasing solubility of tea beverage in cold water. Thus, tannase has been widely used to hydrolyze tea cream in the processing of tea [87]. 

Enzymatic treatment of tea beverage leads to a better color appearance, less cream formation, better taste, mouth feeling, and overall acceptance [86]. Also, the hydrolysis of the main tea phenols epigallocatechin gallate and epicatechin gallate to epigallocatechin and epicatechin, respectively, increases the antioxidant activity of tea beverage [88].

### 4.2. Beverage Clarification

New fruit juices (pomegranate, cranberry, raspberry, etc.) have recently been acclaimed for their health benefits, in particular, for their antioxidant properties. However, the presence of high tannin content in those fruits is responsible for haze and sediment formation, as well as for color, bitterness, and astringency of the juice upon storage. Enzymatic treatment with tannase may be used to improve the quality of these juices [3].

Rout and Banerjee [89] reported the use of tannase for pomegranate juice debittering. Enzymatic treatment resulted in 25% degradation of tannin, while a combination of tannase and gelatin (1 : 1) resulted in 49% of tannin degradation. This treatment has no negative impact on the biochemical and quality attributes of the fruit juice. Hydrolysis by immobilized tannase removed up to 73.6% of the tannin present in Indian gooseberry (*Phyllanthus emblica*) juice. This enzymatic treatment reduced the content of tannin but increased the gallic acid concentration with a minimum reduction in vitamin C (only 2%) [90, 91]. 

Tannase is used as clarifying agent in refreshing drinks with coffee flavor [50], and, recently, a process for the enhancement of the antioxidant properties of coffee by tannase and other enzymes has been patented [92].

Tannase has also been utilized in grape musts and barley worts as a prefermentative treatment, coupled with conventional fining for stabilizing wine and beer [93]. Tannase is employed for the elaboration of acorn wine. Its use in this process favors the production of a better beverage with an alcoholic content of 10%, reducing sugars content of 7% and a pH of 4.0. In this process, tannase produced by an *Aspergillus* strain helps improving the flavor of the beverage [4].

### 4.3. Gallic Acid Production

Gallic acid (3,4,5-trihydroxybenzoic acid) is a phenolic compound and the monomeric unit of the gallotannins and complex tannins. Gallic acid and related compounds possess many potential therapeutic properties including anticancer and antimicrobial properties [94]. Its major application area is in the manufacture of the antibacterial agent trimethoprim. It is also used in leather industry, in manufacturing gallic acid esters, such as propyl gallate, a potent antioxidant utilized as antioxidant in fats and oils, in the manufacture of pyrogallol and as a photosensitive resin in semiconductor production [10]. 

Conventionally, gallic acid is produced by acid hydrolysis of tannins, but this process releases a large amount of toxic effluent that causes environmental hazards [95]. Thus, biotechnological production of gallic acid by tannin fermentation or enzymatic hydrolysis should be preferred. However, these biological methods should be optimized to offer highly productive bioprocesses [96].

Several fungi, bacteria, and yeasts have been used to produce gallic acid from tannin-rich materials with the simultaneous production of tannase. Microbial production of gallic acid from tara (*Caesalpinia spinosa*) [97], sumac (*Rhus coriaria*) [98], myrobalan (*Terminalia chebula*) [99], teri pods (*Caesalpinia digyna*) [100], creosote bush (*Larrea tridentata*), and tar bush (*Flourensia cernua*) [101], among others, has been published. The main difficulty in the development of a successful bioconversion process is the sensitivity of the microorganisms to tannin and the oxidation of the unused tannin [102]. Additionally, gallic acid, released from tannin, can be easily assimilated by microorganisms. However, several strategies such as coculture, strain improvement and optimization of culture conditions have led to the development of fermentation processes with yields higher than 90% of theoretical value [13, 99, 103].

On the other hand, there are only a few papers on the enzymatic production of gallic acid. Battestin and coworkers [104] described the simultaneous production of gallic acid and EGC from an extract of green tea by free *Paecilomyces variotii* tannase. More recently, Curiel et al. [27] reported a process for the enzymatic production of gallic acid. They immobilized a recombinant tannase from *L. plantarum* expressed in *Escherichia coli* then utilized the immobilized enzyme for the hydrolysis of commercial tannic acid. At least 95% of tannic acid was transformed into gallic acid, obtaining an almost pure compound.

### 4.4. Synthesis of Gallic Acid Esters

Tannase, is an enzyme characterized for catalyzing the hydrolysis of gallic acid esters. But, under appropriate conditions, this enzyme can synthesize esters of gallic acid. Toth and Hensler discovered the ability of soluble tannase to produce gallic acid esters. Weetall [105] reported the enzymatic synthesis of a variety of gallic acid esters. He applied an immobilized tannase from *Aspergillus niger* to a solution of gallic acid in different alcohols (C_1_–C_12_) and diols (C_3_–C_6_). Tannase was capable of catalyze the synthesis of esters and diesters of gallic acid, and maximum esterification efficiency was found with alcohol an diol with 4-5 carbon chain. Raab et al. [106] utilized an *A. oryzae *tannase immobilized in Eupergit C for the galloylation of catechins in room temperature ionic liquids (RTIL). RTIL are generally composed of organic cations and inorganic anions and are characterized by not being crystallized at room temperature. The biocatalyzed reactions in ionic liquids have higher selectivity, faster rates, and increased enzyme's thermal stability. 

Mycelium-bound tannase from *Aspergillus niger* has been employed to catalyze the synthesis of propyl gallate in organic solvents. After evaluating the reaction parameters, a maximum molar conversion of 65% was achieved. These results were more promising than those of immobilized pure tannase (49.4% yield) or microencapsulated mycelium-bound tannase (36.2% yield) previously reported by the same research group [107, 108]. Since under optimum fermentation conditions tannase is strongly bound to the mycelium [109], the use of whole cells as biocatalyst could offer several technical and economical advantages such as the avoidance of costly and time-consuming purifications [108].

Another interesting approach is the direct synthesis of propyl gallate by direct transesterification of tannic acid. Sharma and Gupta [110] immobilized an *Aspergillus niger * tannase on Celite and applied it to a solution of commercial tannic acid in n-propanol. After optimizing the process parameters, they obtained a reasonable product yield of about 86%.

### 4.5. Elucidation of Polyphenolic Compounds Structure

Tannase has been widely utilized in research and fine chemistry for the elucidation of polyphenolic compounds structure. For example, Armitage et al. [111] studied the structure of Chinese, Turkish, Sumac, and Tara tannins. They found, after hydrolysis by tannase, that such tannins have a core of glucose and suggested that contains 8-9 galloyl moieties per tannin molecule.

More recently, ellagitannins and complex tannins have attracted the attention of many researchers because of their diverse biological activities and their potential as therapeutic agents. Since tannase selectively hydrolyzes the galloyl residues present in these tannins, it has played an important role in the elucidation of complex polyphenolic compounds. Tanaka et al. [112] reexaminated the structure of cercidinin A, an ellagitannin isolated from the bark of *Cercidiphyllum japonicum*. They utilized a commercial tannase for selectively hydrolyzing the galloyl groups of molecule and deduced the location of the esters on the glucopyranose by two-dimensional NMR spectral analysis. Ivanov and coworkers [113] isolated two catechins from an aqueous ethanol extract of *Bergenia crassifolia* rhizomes [(+)-catechin 3,5-di-*O*-gallate and (+)-catechin 3-*O*-gallate]. These compounds strongly inhibited human pancreatic lipase and exhibited a remarkable free radical-scavenging ability. The structure of these molecules was elucidated using MS NMR before and after degalloylation by tannase.

### 4.6. Animal Feed Preparation

It is well known that high levels of dietary tannins have negative effects on animal nutrition; these effects are related to their capacity to bind macromolecules. Tannins form strong complex with enzymes, minerals, and other nutrients. They are also responsible of a bitter taste, which considerably reduces the feed intake [50]. 

Tannins are ubiquitous in nature and are widely found in feedstuffs, forages, fodders, and agroindustrial wastes, affecting livestock production [114]. Antinutritional effect of tannin could be reduced by a treatment with tannase or tannase-producing microorganism. For example, there are some cultivars of sorghum with high content of tannins. Tannin content could be decreased by an enzymatic treatment, and this material could be used as complement in animal diet [4].

Nuero and Reyes [115] reported the production of an enzymatic extract containing tannase from mycelial wastes of penicillin manufacture. This preparation was applied to several flours used as animal feed (barley, bran, maize, oat, rye, soya, and wheat flour). The enzymatic extract from mycelia waste released similar amounts of reducing sugars from all flours when compared with a commercial enzymatic additive used in animal feeding. These observations indicated that tannase-containing preparation has a high potential as supplements for animal feeding.

### 4.7. Nutritional Improvement of Legume Flours

Legumes are of major nutritional importance, especially in developing countries. Seed legumes have high protein contents, and this protein is of good biological value. However, they also have several antinutritional factors, affecting the digestibility of nutrients [116]. Different thermal and biological processes have been used to reduce the antinutritional factors content, increasing their nutritional value. The flours obtained from the processed legumes can be used as ingredients in food preparations.

Several researchers have proposed the use of tannase alone or in combination with other enzymes for the degradation of some antinutritional factors (tannins) present in legume flours. Dueñas et al. [117] studied the effect of the addition of tannase and other enzymes to a lentils (*Lens culinaris*) flour. They found the production of several phenolic compounds (gallic acid, gallic aldehyde, protocatechuic acid, and quercetin 3-*O*-rutinoside, among others) after the treatment with tannase, the decrease of other phenolics such as catechin, epicatechin, and catechin 3-*O*-glucose, and significant increment on the antioxidant activity. However, further investigations, from the same research group, demonstrated that these biochemical changes cannot be completely attributed to the action of tannase. But during enzymatic hydrolysis with tannase, endogenous enzymes were activated. These enzymes can bring out synergic and/or antagonist effects depending on the structure of phenolic compound [118].

 On the other hand, the application of tannase on pea (*Pisum sativum*) led to a decrease on all the phenolic compounds studied and a reduction of the antioxidant capacity [119]. But, in a different experiment, the hydrolysis of pea flour by tannase led to a significant improvement in daily weight gain of rats. This increment was associated with a higher dietary intake of food and total available sugars [120].

### 4.8. Bioremediation of Tannin-Contaminated Wastewaters

Tannins occur commonly in the effluents derived from several agroindustries. The treatment of this kind of wastewaters is usually difficult because tannins are highly soluble and inhibit the growth of many microorganisms [121]. Tannase can be potentially used for the degradation of tannins in those effluents [50]. 

Several authors have reported the biodegradation of tannin-containing wastewaters using model systems. Kachouri and coworkers [122] studied the biodegradation and decolourisation of olive-mill wastewater by *Aspergillus flavus*. The microorganism removed 58% of color and 46% of the chemical demand of oxygen of the wastewater after 6 days of cultivation. Authors associated this degradation with deconcomitant production of tannase, since no lignin peroxidase nor manganese peroxidase were detected, and laccase activity was much lower than tannase activity. 

More recently, Murugan and Al-Sohaibani [123] reported the use of immobilized tannase from *Aspergillus candidus* for the removal of tannin and the associated color from a tannery effluent. Enzymatic treatment removed about 42% of the tannin content and 20% of the color of tannery wastewater. These findings suggest than tannase or tannase-producing microorganism could be utilized for a pretreatment of tannin-rich wastewaters. However, more research is needed to implement this biological treatment at large scale.

### 4.9. Other Potential Applications of Tannase

Fuel ethanol production from agroindustrial wastes has gained much attention in recent years. When these feed stocks are pretreated for delignification, simple or oligomeric phenolics and derivatives are generated from lignin. These compounds can inhibit the hydrolysis catalyzed by cellulases. Tannase could be utilized for degradation of these oligomeric phenolics and, by doing so, mitigate the inhibition on cellulolysis [124].

Tannase gene and tannase activity could be utilized for the identification of *Staphylococcus lugdunensis* in humans and as an indicator of colon cancer [36]. Tannase has been utilized for the production of molecules with therapeutic applications, such as some esters derived from prunioside A with anti-inflammatory activity [125]. Other potential applications of tannase are found in the manufacture of laundry detergents as an additive, in cosmetology to eliminate the turbidity of plant extracts, and in the leather industry to homogenize tannin preparation for high-grade leather tannins [52, 126].

## 5. Upstream Processing of Tannin Acyl Hydrolase

Despite the many applications of tannase, industrial-scale production is very limited, mainly because of its high production cost. At our knowledge, only a few companies produce and sell tannase [50], and the cost of this enzyme is often much more expensive that other industrial-grade enzymes, even from the same supplier. 

Therefore, many efforts have been made to improve the productions systems. These works include the screening for new tannase-producing microorganisms, the application of novel fermentation systems, the optimization of culture conditions, and the production of the enzyme by recombinant microorganism, among other strategies. 

### 5.1. Tannase Sources

Tannase can be obtained from tannin-rich plants and animal tissues, but, for its industrial production, microbial sources are preferred. Enzymes produced by microorganisms are usually more stable than their counterparts of plant or animal. In addition, the fermentation process can produce large amounts of enzymes in a constant and can be controlled more easily [52]. 

It is well known that tannins inhibit the growth of many microorganisms, but there are species that have developed mechanisms to degrade and use them as sole carbon source. These mechanisms include the production of tannase and other related enzymes [127]. It was previously stated that only a few microorganisms are able to produce tannase. However, it has been identified that more than 70 species produce this enzyme, and the number keeps growing as a result of the continuing search for new sources of this enzyme [128]. Tannase is mostly produced by fungi from the *Aspergillus* and *Penicillium* genus and lactic acid bacteria. 

Several research groups have recently carried out a search for new sources of tannase. These researches are made for finding out microorganisms able to produce higher enzymatic titers or enzymes with desirable properties, such as more stability at a broad range of temperature and pH. 

The most common strategy is to isolate microorganisms from tannin-rich environments and investigate their ability to produce tannase. For example, Murugan et al. [129] isolated 10 morphological different fungal strains from a tannery effluent in India. They isolated the microorganism by serial dilution in PDA slants and investigated for tannase production by a simple plate assay. Selected microorganisms were tested for tannase production under SmF in a stirred tank bioreactor. Pepi and coworkers isolated 3 bacterial strains capable of degrading tannic acid from olive mill waste mixtures. The isolated bacteria, belonging to the *Pantoea* and *Serratia* genus, were able to grow in SmF with tannic acid as sole carbon source and completely degraded a 1% tannin solution within 24 h [9].

 Other common approach is to investigate the tannase presence in microorganisms obtained from culture collections. Pinto and coworkers investigated the tannase activity of 17 wild type and 13 mutant strains of *Aspergillus niger *from a local culture collection in Brazil (EMBRAPA/Food Technology stock collection). They selected the potential tannase producers by their ability to grow in agar plates with tannic acid as sole carbon source. Then the selected microorganisms were utilized for tannase-production under SSF [130]. A similar strategy was developed by the group of Battestin and Macedo [81]. They screened the tannase producing potential of five hundred fungal cultures from the Food Science Department, UNICAMP Culture Collection (Brazil) and identified the best tannase-producing fungus as *Paecilomyces variotii*.

On the other hand, a few researches have isolated microorganism from extreme environments to find extremophile tannases with particular characteristics desired. For example, Cruz-Hernández and coworkers [5] isolated and characterized eleven fungal strains from soil and tannin-rich plants of the Mexican semidesert. These xerophilic fungi were able to produce tannase and degrade high tannin amounts in low-humidity conditions. Kasieczka-Burnecka et al. [12] isolated an Antarctic filamentous fungus from the soil of the King George Island (South Shetlands). This strain (identified as *Verticillium* sp.) produced two psychrophilic tannases with an optimal temperature of 20 and 25°C, respectively.

### 5.2. Production System

Traditionally, industrial production of tannase was carried out exclusively in SmF systems. However, in the recent years a number of investigations have shown the benefits of SSF for production of this and other enzymes. These advantages are higher degrees of activity, increased productivity, extracellular nature of the enzyme, and increased stability to pH and temperature changes. In addition, the SSF allows the construction of more compact reactors with less energy requirements and causing less damage to the environment [52, 131].

Cruz-Hernández et al. [132] evaluated the effect of culture system on the production of tannase by an *Aspergillus niger* strain. They found that enzyme production was about four times higher in SSF compared with SmF. These results are similar to those previously obtained by Aguilar et al. [28, 29] with another strain of *A. niger*. They obtained an activity and productivity at least 2.5 times higher in SSF and associated the low productivity of the SmF to a possible degradation of the enzyme that is not present in SSF. 

Lekha and Lonsane [133] compared the production of tannase in SSF, SmF, and Liquid Surface Fermentation (LSF) by *Aspergillus niger* PKL104 and found that the enzyme production in SSF was about 2.5 and 4.8 times higher than that obtained by SmF and LSF, respectively. In addition, the activity peak reached in SSF was obtained in about half the time required by the other two systems. Results obtained by Rana and Bhat [74] with another *A. niger* strain also showed that the SSF system is better for tannase production; in that case, the maximum yield achieved in SSF was 1.6 times higher than that obtained by SmF; also tannase produced by SSF was more stable at a wide range of temperatures and pHs.

SSF traditionally involves the microbial growth on a moistened natural solid substrate in the absence of free-flowing water. But, in the last years, a second type of SSF system is gaining acceptation. This second system involves the utilization of an inert solid support impregnated with a defined culture media [134]. This culture system facilitates the quantification of biomass, substrate, and products during fermentation and is particularly useful in basic studies on SSF. 

Among the natural supports that have been used for the production of tannase are sugarcane bagasse [135], wheat bran [75], tamarind seed powder [136], palm kernel cake [136], cashew apple bagasse (*Anacardium occidentale*), fruits of *Terminalia chebula* [99], pod cover of *Caesalpinia digyna* [99], ber leaves (*Ziziphus mauritiana*) [137], jamun leaves (*Syzygium cumini*) [137], amla leaves (*Phyllanthus emblica*) [137], jawar leaves (*Sorghum vulgaris*) [137], and Creosote bush leaves (*Larrea tridentata*) [138]. The most commonly used inert support for the production of tannase is the polyurethane foam [28, 29].

There are several papers on the production of tannase in innovative systems. For example, Kar et al. [100] reported the simultaneous production of tannase and gallic acid by *Rhizopus oryzae* in a modified solid-state fermentation system (mSSF). The mSSF was carried out in a Growtek bioreactor and showed several advantages over traditional SSF, including increased production of tannase (1.7 times) and a gallic acid yield almost three times higher. Van de Lagemaat and Pyle [14, 139] developed a prototype of continuous solid-state fermentation (cSSF) for fungal tannase production; the bioreactor achieved a good mix that allowed their operation with a sterile food; however, the rotation resulted in reduced growth and sporulation.

Another interesting approach is the production of tannase by immobilized cells. The immobilization of cells offers many advantages over the utilization of free cells, such as immobilized cell particles are more easily to handle and can be packed in fermenter system for industrial processes; the support materials provide a stabilizing effect on the cellular activities; the enzymes secreted are largely free of cells and cell debris which facilitates downstream processing [140]. Das Mohapatra and coworkers immobilized active cells of *Bacillus licheniformis *in Ca alginate beads and used them for tannase production under semicontinuous fermentation. Tannase production by immobilized cells was about 1.70-fold higher than that obtained by free cells in the same incubation period. The immobilized cells were reutilized over 13 repeated cycles and reached a maximum level at the third cycle [141]. More recently, Darah et al. [140] reported the immobilization of *Aspergillus niger *cells by entrapment in scouring mesh cubes. The immobilization of cells led to a 41.6% increment in tannase production and allowed the reutilization of the cells.

### 5.3. Optimization of Culture Parameters

The tannase production remains a challenge for biotechnologists, due to its high processing cost. In the previous sections, it was mentioned that different research groups have devoted considerable efforts in finding new sources of tannase and designing better production systems. However, the conditions for obtaining the maximal production of the enzyme depend on two factors: the system utilized and the source of the enzyme. Thus, recently there have been published a number of papers dealing with the optimization of solid-state fermentation systems for the production of tannase [16–18, 142–145]. In most of those cases, optimization was carried out by changing one independent variable at a time while fixing all the others at a certain level. This method is very time consuming and requires a large number of experiments to determine the optimum levels. Also, this approach does not include the interactive effects among the variables and is, therefore, unreliable. Thus, an statistical optimization is preferred [146].

Raaman et al. optimized the extracellular tannase production by *Paecilomyces variotii *in SSF changing one factor at time and obtained an increase of 1.23-fold in enzyme production. Das Mohapatra and coworkers reported the optimization of tannase production by *Bacillus licheniformis* in SmF using Taguchi's methodology. They reached a 2.18-fold increment in tannase [19]. 

Sharma and coworkers [147] optimized tannase production by *Aspergillus niger *using response surface methodology and obtained 2.01-fold improvement on enzyme production. Naidu et al. [148] optimized the tannase production by *Aspergillus foetidus* in SmF utilizing subsequently two statistical methods: first, a Plackett-Burman design was employed for find out the key factors for tannase production, then these factors were optimized by response surface methodology using a central composite design. With this strategy, they obtained a twofold increase in tannase activity in comparison to the medium optimized by the conventional one factor at a time method. In addition, the number of nutrients for the production of tannase was reduced from ten to four after optimization.

### 5.4. Recombinant Tannases

Because of the many technical difficulties involved in production of tannase using traditional technologies; in recent years, there has been a great interest for the production of this enzyme by recombinant microorganisms. In 1996, Hatamoto et al. cloned and sequenced for the first time the gene encoding tannase from *Aspergillus oryzae*. This gene was further expressed in a tannase low-producing *A. oryzae* strain. Tannase production in transformants was at least threefold higher than that in the wild strain. Southern blotting confirmed that the increase in the tannase level was due to the presence of additional copies of the tannase gene in the genome of the transformants [43]. Based on these results, Albertse sequenced the tannase gene from four tannase-producing *Aspergillus* species. He cloned and inserted the tannase gene from *A. oryzae* in *Saccharomyces cerevisiae. *However, the biological activity of the recombinant tannase was expressed at very low level, probably due to differences in posttranslational modifications in the heterologous system with respect to the wild microorganism [24].

Zhong and coworkers successfully cloned and sequenced a tannase gene from *Aspergillus oryzae* in the methylotrophic yeast *Pichia pastoris*. The inserted gene was under the control of *AOX1* promoter and inframe with *α*-factor signal sequence. Large amounts of extracellular tannase (7000 U/L) were obtained with transformed yeast in fedbatch SmF system, using glycerol as carbon source and methanol as tannase inducer [23]. Yu and coworkers achieved efficient intracellular expression of *A. oryzae* tannase in *P. pastoris* under the control of the *AOX1* promoter. The recombinant *P. pastoris* were used to synthesize propyl gallate in organic solvent, and the yield of propyl gallate was 53%. 

More recently, Böer and coworkers reported the overexpression of the tannase gene *ATAN1* in the auxotrophic mutant strain *Arxula adeninivorans* G1212. The *ATAN1* gene was under the control of the strong, constitutive *TEF1* promoter, and its product was directed into the secretion pathway by its own 28-amino acid secretion sequence. The recombinant *A. adeninivorans* produced levels of up to 400 U/L when grown in glucose medium in shake flasks. Whereas the wild type strain LS3 secreted amounts of tannase equivalent to 100 U/L under inducing conditions [21].

On the other hand, Iwamoto et al. [40] identified the tannase-encoding gene from *Lactobacillus plantarum*. The tannase gene was cloned and hyperexpressed in *Escherichia coli*; the recombinant tannase was further purified through several chromatographic steps and biochemically characterized. For improving the purification of the recombinant *L. plantarum* tannase, Curiel and coworkers used the vector pURI3 to express the tannase gene in *E. coli*. The pURI3 vector was created using the pT7-7 vector as template and contained an amino-terminal His-tag that allowed the purification of the recombinant protein directly from the crude cell extracts in a single-step procedure [20]. 

Moreover, Yao et al. [149] isolated a novel gene encoding tannase (*tan410*) from a cotton field metagenomic library by functional screening. The *tan410* gene was cloned and expressed in *E. coli *as an N-terminal His-tag fusion protein using pET-28a expression system under the control of T7 *lac* promoter. The recombinant tannase was purified and characterized, and it was found to have interesting properties for biotechnological applications.

## 6. Downstream Processing of Tannase

In the previous section, it was established the need for improvement of the tannase production processes (upstream processing). Another important aspect on industrial tannase manufacture is the downstream processing operations.

Downstream processing of proteins involves all the operations after the production process that is, recovery, concentration, and purification. The number and the type of these operations depend on the nature of the raw material and the necessary purity in the final product. Anyway, during the downstream processing, it should not lose more of the product than that absolutely necessary [150]. 

In the case of tannase, there are a number of papers describing different protocols for enzyme recovery, concentration, and purification. Most of them are focused in the purification of tannase for analytical purposes, such as biochemical or molecular characterization. These protocols are useful for obtaining high-purity protein, but the operations utilized are slow, expensive, and inefficient for industrial purposes. The degree of purity required for industrial enzymes is much lower. It is often sufficient that the purity of the enzyme ensures the stability and the absence of undesirable reactions. In other cases, it also must be guaranted the product safety. Thus, it should be designed specific protocols for enzyme purification for industrial applications. In this section, the main advances in downstream processing of tannase will be discussed. 

### 6.1. Recovery

The method for tannase recovery depends on the production system, the microorganism used, and the time of extraction. In SSF, tannase is produced mainly extracellularly and recovery is easily achieved, simply add two or three volumes of extractant (distilled water or buffer) mixing and compression, to obtain an enzyme extract. In the case of the SmF, the location of the enzyme depends on the microorganism and the incubation time [3].

In SmF, fungi produce intracellular tannase during the first hours of incubation. At this stage, enzyme recovery implies cell disruption and extraction with an appropriate agent. Tannase is lately excreted, making its recovery easy. However, at the moment of maximal production, 75% of the enzyme remains bound on the mycelium [59]. On the other hand, most of the bacterial strains produce extracellular tannase in SmF. However, recently, it has been found an interesting cell-associated tannase from *Serratia ficaria* produced in SmF [8].

Cell disruption may be achieved by chemical, enzymatic, or mechanical procedures. Barthomeuf et al. [109] reported that the classical physical and chemical methods were unable to release the tannase attached to the *Aspergillus niger* mycelium. No more than 5% of the enzyme was recovered by grinding mycelium with sand or glass beads or pulverizing with a homogenizer; osmotic shocks or ultrasonic waves in various buffers neither were productive. For the efficient recovery of this mycelium-bound tannase, they proposed an enzymatic hydrolysis of cell walls using a chitinase from *Streptomyces griseus* followed by reverse micellar extraction. This protocol resulted in a recovery of 43% active enzyme.

On the other hand, Bhardwaj and coworkers found that tannase extraction from the mycelium of *Aspergillus niger* strongly depends on the pH of the extractant agent and that maximum enzyme recovery is reached at pH 5.5. In addition, they found that homogenization and detergent pretreatments do not have any remarkable effect on the extraction of tannase [151].

### 6.2. Concentration

Water is considered as a major contaminant in enzyme preparations, particularly in those that are excreted to a fermentation broth. Thus, protein concentration operations are often the first step in protein purification protocols.

Traditionally, tannase concentration has been carried out by precipitation with salts or solvents. These techniques are simple, rapid, economical, and do not require sophisticated equipment. But they often lead to low-recovery yield of enzymatic activity due to irreversible denaturation of protein. For example, Battestin and Macedo concentrated a *Paecilomyces variotii* tannase by sulfate precipitation; they obtained a 1.9-fold purification with a recovery yield of 34% [81]. Lekha and Lonsane [133] precipitated an *Aspergillus niger *GH1 with acetone. They obtained a 6.2-fold purification but with a low recovery yield of 28%.

Another method utilized for downstream processing of tannase is the concentration with polyethylene glycol. The enzymatic extract is put into a dialysis membrane bag and placed on a layer of PEG 6000 [76]. This technique allows a rapid and efficient concentration and does not require any specialized equipment. But the method has several complications, especially for scaling up. Enemuor and Odibo used a variation of this methodology for tannase concentration. They assayed the dialysis of an enzymatic extract against 6 M sucrose and obtained a 23-fold concentration. However, the recovery yield was extremely low (1.9%) [153]. 

On the other hand, ultrafiltration has been used to efficiently concentrate tannase. This technique is rapid and leads to a high-recovery yield. In addition, when using appropriate membranes, ultrafiltration can be used for simultaneous concentration and purification of proteins. It has the disadvantage of requiring specialized equipment but then can be used on a large-scale downstream processing. Sharma and coworkers reported the use of ultrafiltration for the concentration of a *Paecilomyces variotii* tannase. They utilized a 100 kDa MWCO membrane and concentrated 5.4 times the crude extract. Using this methodology, they achieved a 5.2-fold purification with a recovery yield of 97% [32].

### 6.3. Purification

The purification process is one of the less developed aspects on tannase. Most of the published purification protocols consist on multistep procedures able to obtain a highly purified enzyme but with a low-recovery yield.  Table 4 summarizes several protocols utilized for this aim.

Probably the most common strategy used for the purification of tannase based on protein concentration followed by ion exchange and/or gel filtration chromatography [7, 151, 152]. Sharma and coworkers purified to homogeneity the intracellular tannase from *Aspergillus niger* MTCC 2425. They designed a protocol consisting in protein concentration with polyethylene glycol followed by ionic exchange chromatography in a DEAE-cellulose column and gel filtration using a Sephadex G-150 column. This strategy led to a high-purity enzyme but with a low-recovery yield (2.7%) [76]. 

The efficiency of a purification protocol mainly depends on the number and the type of operations utilized. But this is not the only important factor; the order of operations can drastically affect the process performance. Bhardwaj et al. [151] improved the above-mentioned protocol by changing the order of chromatographic steps. This strategy helped to overcome the problem of binding of fungal pigment to the matrix of DEAE column; since the fungal pigment did not bind to Sephadex G-150 and was eluted after the proteins, the performance of ionic exchange chromatography was improved.

Other authors have applied more sophisticated technologies for tannase purification. Beverini and Metche [58] purified two isoforms of tannase from a commercial enzyme powder prepared from culture broth of *Aspergillus oryzae*. They subsequently used precipitation with acetone, gel filtration on Sephadex G-50 and Biogel P-300 columns, and affinity chromatography on Con-A Ultrogel. Ramírez-Coronel and coworkers purified an *A. niger* tannase by preparative isoelectric focusing followed by ionic exchange (MonoQ column) and gelfiltration chromatography (Sephadex G-100) [42].

Another interesting approach is related to the purification of recombinant tannases. Zhong and coworkers reported that the recombinant tannase from *Aspergillus oryzae* expressed in *Pichia pastoris* was easily purified to homogeneity. They purified the enzyme by ultrafiltration followed by ionic exchange and obtained 72 mg of pure protein from 1000 mL of culture broth (50 U/mg) with a recovery yield about 50% [23]. Curiel et al. described a high-yield protocol for the purification of a recombinant *Lactobacillus plantarum* tannase expressed in *Escherichia coli*. The protein was cloned containing an affinity hexa-His tag, this allowed to purify the recombinant tannase directly from the crude extract using a His-Trap-FF chelating column. By this strategy, they obtained high amounts of pure recombinant tannase (17 mg/L) by a one-step affinity procedure [20].

On the other hand, a few authors have developed strategies for downstream processing of tannase based on rapid, economic, and easily scalable unit operations. Gupta and coworkers developed a rapid procedure for partial purification of extracellular tannase based on the precipitation of protein with a combination of tannic acid and PEG-6000. They obtained eightfold purification by a twostep protocol and a recovery yield about 50% [25]. Naidu et al. Partially purified an *Aspergillus oryzae* by a procedure involving ammonium precipitation and liquid-liquid extraction in aqueous two-phase systems [26]. This protocol led to a purification fold of 2.70 and yield of 82.0%. These strategies are simple and convenient. Therefore, they have potential application in the processing of tannase for industrial use, where absolute purity is not necessary, or in the early stages of a total purification.

## 7. Concluding Remarks

Tannase is an enzyme with a number of potential applications in biotechnology. However, many of these applications have been missed due to the high cost of the enzyme. In this regard, in recent years, it has been made significant progress for improving the upstream and downstream processing of tannase. Several research groups have developed economic and ecofriendly processes for the production and purification of the enzyme at laboratory level. On the other hand, the use of modern techniques of molecular biology has allowed the development of highly efficient processes for production and recovery of the enzyme, and this appear to be the trend to continue in the coming years. The results have been positive, but more research is needed on basic and applied aspects of tannase, such as regulation of the enzyme, metagenomics, new expression systems, design of new bioprocesses using emerging large-scale cultivation technologies, efficient and cost-effective downstream processing and design of new applications for the enzyme such as the production of antioxidants from waste materials or the bioremediation of tannery effluents.

## Figures and Tables

**Table 1:** Structural properties of some characterized tannases.

Microorganism	Culture system	MW (kDa)	Subunits	Glycosylation (%)	Reference
*Arxula adeninivorans*	SmF	320	4 × 80	31.2	[21]
*Aspergillus awamori*	SmF	230	6 × 37.8	8.0	[37]
*Aspergillus niger*	SSF	225	50 + 75 + 100	n.d.	[31]
*Aspergillus oryzae*	SmF	310	4 × (30 + 33)	22.7	[43]
*Candida *sp.	SmF	250	2 × 120	64	[67]
*Enterobacter *sp.	SmF	90	1 × 90	0	[39]
*Lactobacillus plantarum*	SmF	50	1 × 50	0	[40]
*Quercus robur*	Plant	150	2 × 75	n.d.	[56]
	leaves	300	4 × 75		
*Selenomonas ruminantium*	SmF	59	1 × 59	0	[68]

n.d.: Not determined.

**Table 2:** Physicochemical properties of some characterized tannases.

Microorganism	Substrate	Temperature optimum (°C)	Stability temperature (°C)	pH optimum	Stability pH	pI	*K_M_* (mM)	*V*_Max_ (*μ*mol/min·mg)	Reference
*Arxula adeninivorans*	Methyl gallate	40	≤50	6.0	5.0–7.0	n.d.	4.4	n.d.	[21]
*Aspergillus awamori*	Methyl gallate	30	≤30	2.0, 8.0	2.0	4.4	1.9	830	[37]
*Aspergillus flavus*	Tannic acid	50–60	≤60	5–5.5	5.0–5.5	n.d.	0.05	n.d.	[71, 73]
*Aspergillus niger*	Methyl gallate	30	≤60	5.0–6.5	4.5–6.5	n.d.	0.6	5	[74]
*Aspergillus niger* Aa20	Tannic acid	60–70	≤90	6.0	3.5–7.0	3.8	n.d.	n.d.	[42]
*Aspergillus niger* ATCC 16620	Methyl gallate	30–40	≤65	6.0	4.0–8.0	n.d.	103	4.25	[75]
*Aspergillus niger* GH1	Methyl gallate	60	≤50	6.0	4.0–6.0	3.5	0.041	11.03	[31]
*Aspergillus niger *LCF8	Tannic acid	35	≤45	6.0	3.5–8.0	4.3	n.d.	n.d.	[59]
*Aspergillus niger *van Tieghem	Methyl gallate	60	≤60	6	4.5–7.5	n.d.	0.2	5	[76]
*Aspergillus oryzae*	Tannic acid	40	≤45	5.5	4.5–6.0	n.d.	7.35	83	[77]
*Candida* sp.	Tannic acid	50	≤70	6.0	3.5–7.5	n.d.	n.d.	n.d.	[78]
*Lactobacillus plantarum*	Methyl gallate	40	≤45	8.0	7.5–9.0	n.d.	0.62	n.d.	[40]
*Paecilomyces variotii*	Tannic acid	55	≤55	5.5	4.5–6.5	n.d.	6.1 × 10^−4^	5.6	[34]
*Penicillium variable*	Tannic acid	50	≤80	5.0	3.0–8.0	n.d.	32	1.11	[32]
*Verticillium* sp. (TAH 1)	Tannic acid	25	≤30	5.5	4.5–6.0	5.8	1.05	n.d.	[12]
*Verticillium* sp. (TAH 2)	Tannic acid	20	≤30	5.5	5.0–7.5	6.2	1.05	n.d.	[12]

n.d.: Not determined.

**Table 3:** Selected patents on tannase application published during the last 10 years.

Year	Assignee	Title	Patent no.
2001	Quest International Nederland	Process for the production of beer having improved flavour stability.	EP 1122303
2002	Unicafe Inc.	Tea extracts stabilized for long-term preservation and method of producing same.	USP 6,365,219
2002	Purdue Research Foundation Pharmanex, Inc.	Tea catechin formulations and processes for making same.	USP 6,428,818
2004	University of South Florida	Vasodilating compound and method of use.	USP 6,706,756
2004	Purdue Research Foundation	Compositions based on vanilloid-catechin synergies for prevention and treatment of cancer.	USP 6,759,064
2004	Lipton, division of Conopco, Inc.	Cold brew tea.	USP 6,780,454
2004	Kyowa Hakko Kogyo Co., Ltd.	Process for purification of proanthocyanidin oligomer.	USP 6,800,433
2004	Lipton, division of Conopco, Inc.	Cold water infusing leaf tea.	USP 6,833,144
2006	Nestec S A	Soluble coffee product.	EP 1726213
2006	Eisai Co., Ltd.	Diagnostic agent and test method for colon cancer using tannase as index.	USP 7,090,997
2006	Unilever Bestfoods	Black tea manufacture.	USP 7,108,877
2007	Eisai R&D Man Co. Ltd.	Novel tannase gene and protein thereof.	EP 1837400
2008	Probelte Pharma S A	Process for preparing pomegranate extracts.	EP 1967079
2008	Novozymes, Inc.	Methods for degrading lignocellulosic materials.	USP 7,354,743
2008	The Procter & Gamble Company	Foam-generating kit containing a foam-generating dispenser and a composition containing a high level of surfactant.	USP 7,402,554
2008	Novozymes, Inc.	Methods for degrading or converting plant cell wall polysaccharides.	USP 7,413,882
2009	Kirin Brewery	Method of enzymatically treating green tea leaves.	EP 2036440
2009	Kao Corp.	Beverage packed in foam container.	EP 2036446
2009	Kao Corp.	Green tea drink packed in container.	EP 2098121
2009	Colgate-Palmolive Co.	Antiplaque oral composition containing enzymes and cyclodextrins.	USP 7,601,338
2009	Novozymes, Inc.	Methods for enhancing the degradation or conversion of cellulosic material.	USP 7,608,689
2010	Kao Corp.	Process for producing purified tea extract.	EP 2225952
2010	University of California	Method for lowering blood pressure in prehypertensive individuals and/or individuals with metabolic syndrome.	USP 7,651,707
2010	J.M. Huber Corporation	High-cleaning silica materials and dentifrice containing such ones.	USP 7,670,593
2010	Novozymes, Inc.	Polypeptides having cellulolytic enhancing activity and nucleic acids encoding the same.	USP 7,741,466
2010	Constellation Brands, Inc.	Grape extract, dietary supplement thereof, and processes therefore.	USP 7,767,235
2011	Taiyo Kagaku Co., Ltd	Composition for inhibiting thrombosis.	USP 7,914,830
2011	Danisco US Inc.	Polyol oxidases.	USP 7,919,295

**Table 4:** Summary of protocols for tannase purification.

Source	Production system	Extra-/intra-cellular	Operations	Purification factor	Recovery yield (%)	Reference
*Aspergillus awamori*	SmF	Extracellular	Aluminum oxide treatment, ultrafiltration (30 and 100 kDa), and gel filtration (Sephadex G-200).	6.32	0.51	[37]
*Aspergillus flavus*	SmF	Intracellular	Ammonium sulfate and tannic acid precipitation, ionic exchange (DEAE-cellulose), gel filtration chromatography (Sephadex G-200), and acetone fractionation.	31	49	[73]
*Aspergillus foetidus*	SmF	Extracellular	Ammonium sulfate precipitation and ATPS extraction	2.7	82.5	[26]
*Aspergillus heteromorphus*	SmF	Extracellular	Ammonium sulfate precipitation and ionic exchange chromatography (DEAE-cellulose)	39.7	19.3	[7]
*Aspergillus niger*	SmF	Intracellular	Ultrafiltration (100 and 200 kDa) and high-pressure size-exclusion chromatography (SW 300).	29.1	15.1	[59]
*Aspergillus niger*	SmF	Intracellular	Concentration with PEG 6000, ionic exchange (DEAE-Sephadex), and gel filtration chromatography (Sephadex G-150).	29	2.7	[76]
*Aspergillus niger*	SmF	Intracellular	Gel filtration (Sephadex G-150) and ionic exchange chromatography (DEAE-Sephadex).	51	20	[151]
*Aspergillus niger*	SSF	Extracellular	Ultrafiltration (1 kDa), ionic exchange (DEAE-cellulose), and gel filtration chromatography (Sephacryl S-300).	46	0.3	[31]
*Aspergillus oryzae*	SmF	Extracellular	Ionic exchange (DEAE-Sephadex), gel filtration (Sephadex G100), ultracentrifugation.	12.8	11.5	[69]
*Candida* sp.	SmF	Extracellular	Pervaporation, rivanol precipitation, ionic exchange (ECTEOLA-cellulose), ultrafiltration, and gel filtration chromatography (Sepharose 6B and Sephadex G200).	613	7.3	[78]
*Enterobacter *sp.	SmF	Intracellular	Ammonium sulfate precipitation, ionic exchange (DEAE-cellulose) and gel filtration chromatography (Sephadex G-100).	162	7.1	[39]
*Lactobacillus plantarum* in *E. coli *	SmF	Intracellular	Ammonium sulfate precipitation and three ionic exchange chromatographic steps (Q Sepharose, Hydroxyapatite, and Mono Q columns).	11.2	4.8	[40]
*Lactobacillus plantarum* in *E. coli *	SmF	Intracellular	Affinity chromatography (poorly activated nickel chelate supports).	15	95	[27]
*Paecilomyces variotii*	SmF	Extracellular	Activated charcoal treatment, ammonium sulfate precipitation, ionic exchange (DEAE-cellulose), and gel filtration chromatography (Sephadex G-200).	30.5	17.6	[152]
*Paecilomyces variotii*	SSF	Extracellular	Ammonium sulfate precipitation and ionic exchange chromatography (DEAE-Sepharose).	19.3	3	[34]
*Penicillium chrysogenum*	SmF	Intracellular	Ammonium sulfate precipitation, ionic exchange (DEAE-cellulose), and gel filtration chromatography (Sephadex G-200).	24	18.5	[80]
*Penicillium variable*	SmF	Extracellular	Ultrafiltration (100 kDa) and gel filtration chromatography (Sephadex G-200).	135	91	[32]
*Verticillium* sp. (TAH 1)	SmF	Extracellular	Ammonium sulfate precipitation and ionic exchange chromatography (DEAE-cellulose).	7.9	1.6	[12]
*Verticillium* sp. (TAH 2)	SmF	Extracellular	Ammonium sulfate precipitation and ionic exchange chromatography (DEAE-cellulose).	10.5	0.9	[12]
